# Hypotension in Severe Dimethoate Self-Poisoning

**DOI:** 10.1080/15563650802172063

**Published:** 2008-11-11

**Authors:** James Davies, Darren Roberts, Peter Eyer, Nick Buckley, Michael Eddleston

**Affiliations:** 1 Department of Critical Care Medicine, Guy’s and St Thomas’ Hospital, London, UK; 2 Medical School, Australian National University, Canberra, Australia, and the South Asian Clinical Toxicology Research Collaboration, Sri Lanka; 3 Walther Straub Institute of Pharmacology and Toxicology, Munich, Germany; 4 Scottish Poisons Information Bureau, Royal Infirmary, and Clinical Pharmacology Unit, University of Edinburgh, Edinburgh, UK

**Keywords:** Acute poisoning, Organophosphate insecticides, Insecticides

## Abstract

**Introduction:**

Acute self-poisoning with the organophosphorus (OP) pesticide dimethoate has a human case fatality three-fold higher than poisoning with chlorpyrifos despite similar animal toxicity. The typical clinical presentation of severe dimethoate poisoning is quite distinct from that of chlorpyrifos and other OP pesticides: many patients present with hypotension that progresses to shock and death within 12–48 h post-ingestion. The pathophysiology of this syndrome is not clear.

**Case reports:**

We present here three patients with proven severe dimethoate poisoning. Clinically, all had inappropriate peripheral vasodilatation and profound hypotension on presentation, which progressed despite treatment with atropine, i.v. fluids, pralidoxime chloride, and inotropes. All died 2.5–32 h post-admission. Continuous cardiac monitoring and quantification of troponin T provided little evidence for a primary cardiotoxic effect of dimethoate.

**Conclusion:**

Severe dimethoate self-poisoning causes a syndrome characterized by marked hypotension with progression to distributive shock and death despite standard treatments. A lack of cardiotoxicity until just before death suggests that the mechanism is of OP-induced low systemic vascular resistance (SVR). Further invasive studies of cardiac function and SVR, and post-mortem histology, are required to better describe this syndrome and to establish the role of vasopressors and high-dose atropine in therapy.

## Introduction

Self-poisoning with organophosphorus (OP) insecticides is a major clinical problem across rural Asia (1), killing an estimated 200,000 people every year (2,3). Most deaths result from respiratory failure, because of the acute cholinergic crisis, complications of pre-hospital pesticide aspiration, and late onset neuromuscular junction failure (4). However, in a large cohort of OP pesticide-poisoned patients (5), we have noted patients with dimethoate poisoning on adequate ventilatory support dying from cardiovascular complications, in particular severe hypotension. Previous reports on cardiac complications of OP poisoning have focused on dysrhythmias (6–11) and hypertension (12,13) with hypotensive effects being relatively less frequently reported(14–17).

Hypotension is a particularly important cause of death in dimethoate poisoning. Over 40% of patients dying from dimethoate poisoning present with a systolic blood pressure (BP) of less than 80 mmHg (compared to 5% of patients dying from chlorpyrifos poisoning) (18). Such patients have a case fatality in excess of 80%. In this article, we describe the clinical features of severe dimethoate poisoning in three patients treated in an under-resourced rural district hospital in Sri Lanka.

## Case Reports

### Case 1

**Fig. 1:**
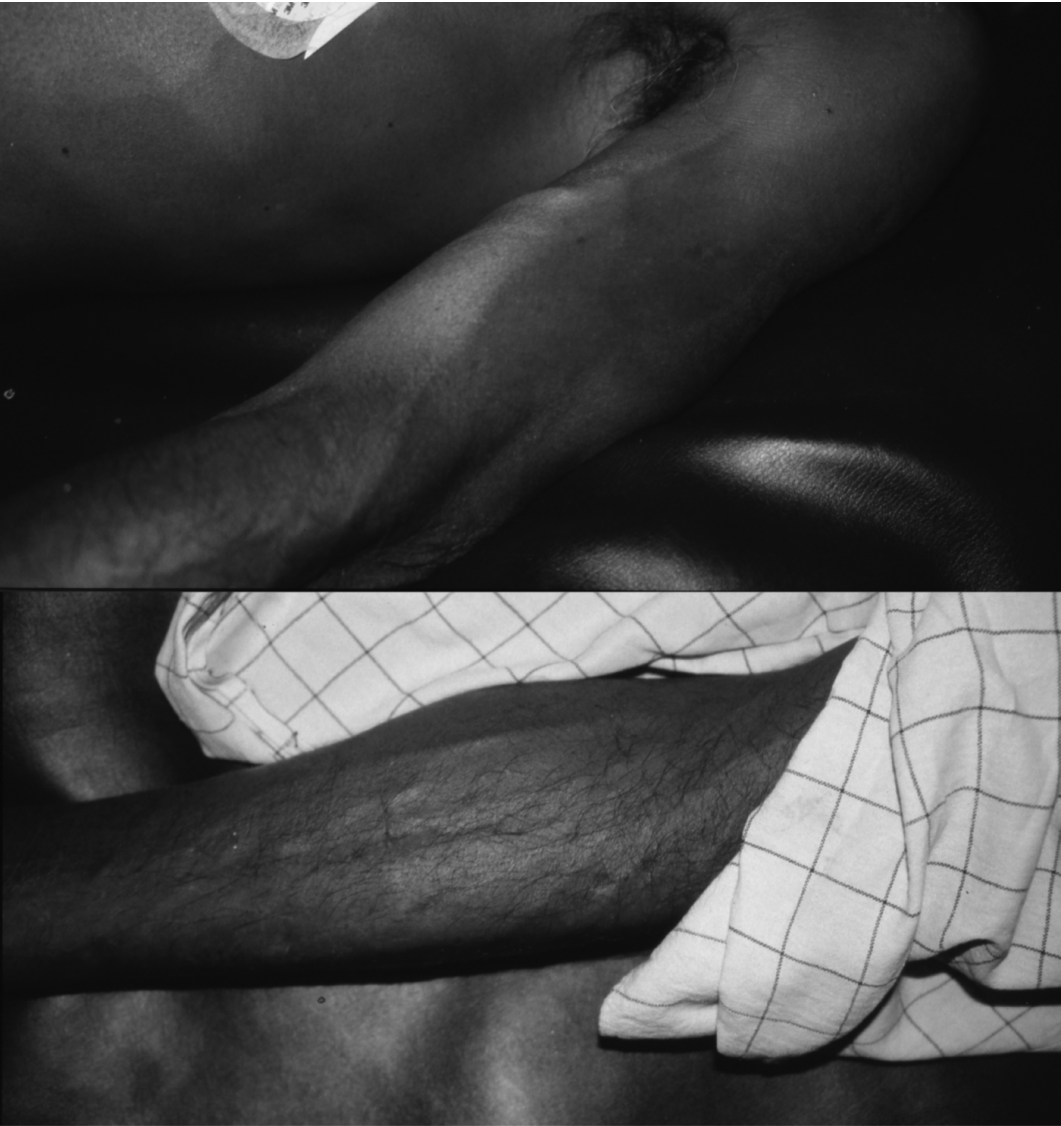
Peripheral vasodilatation in patients 1 and 2 while hypotensive on admission. Both photographs were taken when the patients were supine with BPs of 85/35 mmHg (top, case 1) and 55/ 30 mmHg (bottom, case 2), respectively.

A 55-year-old male presented with cholinergic features after intentionally ingesting an unknown quantity of dimethoate with alcohol. On arrival, he had a pulse of 60 beats/min, blood pressure (BP) 90/60 mmHg, respiratory rate of 5 breaths/ min, and Glasgow Coma Scale (GCS) 3/15. Examination revealed a hyperdynamic pulse and apex beat, normal heart sounds, marked peripheral vasodilatation (Fig. 1), and normal capillary refill time. Widespread wheeze and crepitations were present in both lungs, and pulse oximetry showed an oxygen saturation of 60% despite oxygen at 10 L/min. He was intubated, ventilated, and given boluses of atropine (to a total of 8 mg), and his oxygen saturation improved to 96% on 6 L/min of oxygen. An atropine infusion was then started at 0.5 mg/h, and he received pralidoxime chloride 1 g i.v. followed by 1 g q6h. Two hours after admission, he had normal breath sounds on auscultation and clear lung fields on chest radiograph.

**Fig. 2:**
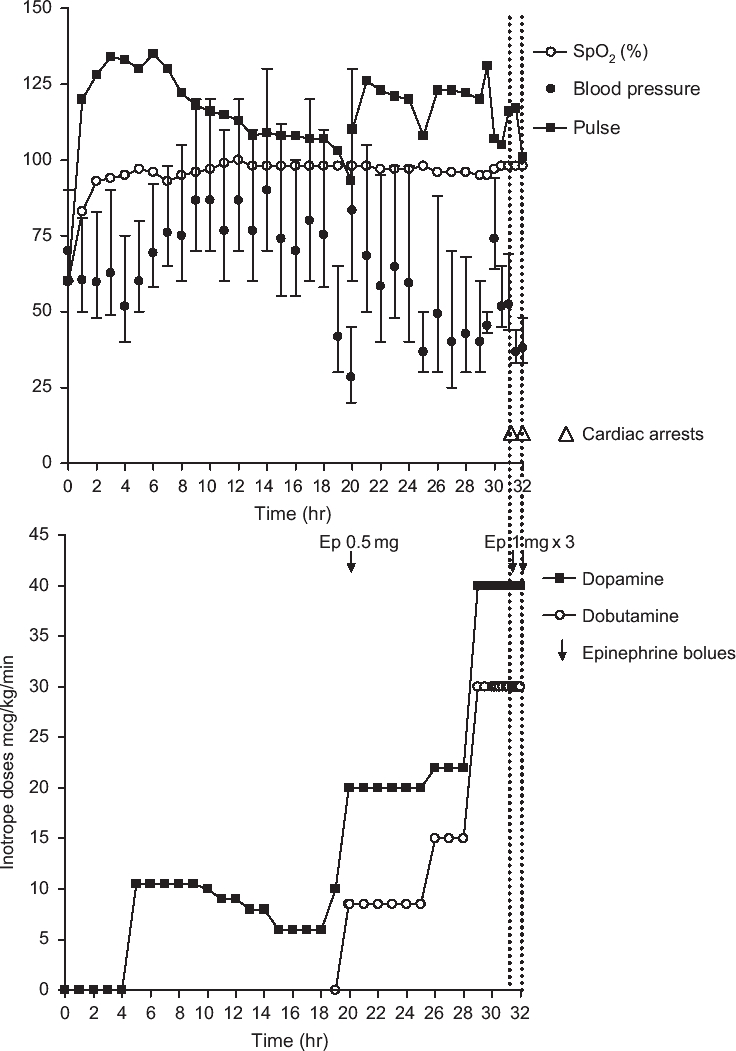
Clinical course of patient 1. The top half of the figure shows heart rate, mean arterial pressure (•) together with systolic and diastolic arterial pressures (↕), and oxygen saturations. The bottom half of the figure shows the infusion rates of both dopamine and dobutamine.

He was commenced on dopamine 10 mcg/kg/min 5 h after admission when his BP became refractory to further fluid challenges with normal saline (Fig. 2). Nineteen hours post-admission, his BP deteriorated to 45/20 mmHg despite doubling the dopamine to 20 mcg/kg/min. He was given 500 mcg epinephrine and started on dobutamine at 10 mcg/kg/min in addition to dopamine (norepinephrine and vasopressin were not available in the hospital). There was a clear response in BP to the addition of dobutamine. By this time, he had developed marked subconjunctival and peripheral edema. His urine output remained >50 mL/h apart from the first 2 h after admission.

Six hours later, his BP fell again despite further fluid challenges totaling 4 L of 0.9% saline plus 10 mg of atropine and increased infusions of dopamine (30 mcg/kg/min) and dobutamine (40 mcg/kg/min). The decline was irreversible and he had a pulseless electrical activity (PEA) cardiac arrest 32 h post-admission from which he did not recover despite administration of epinephrine and atropine.

Blood samples taken 22 h after admission showed the creatine kinase to be elevated (12,640 U/L; Table 1); this was accompanied by rises in aspartate aminotransferase and lactate dehydrogenase but not troponin T. Renal biochemistry was normal. His cardiac rhythm was continuously recorded using a Holter monitor. It showed a sinus tachycardia throughout, with normal complexes up until about 30 min prior to his final cardiac arrest, when there was evidence of T wave inversion and ST depression.

**Table 1:** Dimethoate concentration, butyrylcholinesterase activity, and cardiac enzymes in the three study patients

Case	1		2	3
Time after admission	40 min	22 h	45 min	30 min
Dimethoate concentration (μM)	1,177	438	1,670	1,619
BuChE (mU/mL)	455	134	431	442
CK (U/L; RR 40–180)	43	12,640	109	ND
Tn T (ng/mL; RR <0.09)	0.00	0.03	0.00	ND

BuChE, butyrylcholinesterase (plasma pseudocholinesterase); CK, creatine kinase; ND, not done; RR, reference range; Tn T, troponin T.

Plasma was separated from an EDTA blood sample and frozen at –20°C. BuChE activity was assayed as described (30,31). The median (IQR) BuChE activity in this population is 4,500 (3,200–5,200) mU/mL (Eyer, unpublished). Plasma dimethoate concentrations were quantified by reversed phase HPLC and UV detection (lower limit of quantitation 1 nmol/mL plasma). CK and Tn T were assayed in a clinical laboratory.

### Case 2

A 50-year-old male presented 3.5 h after ingesting an unknown quantity of dimethoate showing cholinergic features. He was rapidly intubated but immediately had a PEA cardiac arrest from which he was resuscitated with 3 mg atro-pine and 1 mg epinephrine. His ECG then showed sinus rhythm with ventricular ectopics. On subsequent examination, he had marked peripheral vasodilatation with distended superficial veins (Fig. 1), normal heart sounds, and an undis-placed apex beat. Auscultation of the lungs revealed bilateral wheeze and crepitations that improved with atropine. His oxygen saturation was normal following atropine and entrained oxygen at 6 L/min within half an hour of his initial cardiac arrest.

Three hours after admission he had a second PEA cardiac arrest from which he could not be resuscitated despite boluses of epinephrine and atropine. Prior to this, we were unable to maintain his BP above 60/35 mmHg despite administering 4 L 0.9% saline, 13 mg of atropine, and dopamine at 70 mcg/ kg/min. He had continued to pass urine during this time.

### Case 3

A 54-year-old male presented 2 h after intentionally ingesting an unknown amount of dimethoate. On arrival, he showed cholinergic features, had a pulse of 41 beats/min, BP 70/40 mmHg, GCS 3/15, pinpoint pupils, and respiratory rate of 6 breaths/min. He was given oxygen and atropine, and rapidly intubated. He responded poorly to atropine (total >200 mg, in boluses of up to 50 mg), becoming more hypotensive and bradycardic, until he had a PEA cardiac arrest in sinus rhythm 30 min after admission. He was resuscitated with boluses of epinephrine totaling 4 mg and given pralidoxime chloride 1 g.

Over the next 2 h his BP and heart rate fell on several occasions and he was given a total of 18 mg epinephrine (equivalent to around 3.3 mcg/kg/min). His response to increasing doses of epinephrine diminished and he died from a PEA cardiac arrest 2.5 h post-admission.

## Discussion

We present here three cases of proven severe dimethoate self-poisoning that resulted in progressive hypotension and death despite administration of standard antidotes, fluid resuscitation, large doses of inotrope/vasopressor agents, and adequate ventilatory support. Because of the lack of evidence for benefit (19,20), gastric decontamination was not performed for any patient. None of the patients had any previous history of cardiac disease or co-morbidity. These three patients provide more detailed description of the hypotensive syndrome previously reported (5). Such a clear clinical syndrome has not been reported for other OPs and its pathophysiology is unclear.

We have previously reported two cases of OP-induced hypotension with substantially reduced systemic vascular resistance (SVR) after dimethoate or fenthion poisoning (14). However, both patients had pre-existing vascular disease. Other authors have reported four patients (two with malathion and two with fenitrothion) that died from hypotensive shock (16). Further, a patient with severe hypotension after combined malathion and fenitrothion poisoning was successfully treated with extracorporeal cardiopulmonary support (15). The responsible OP was not confirmed analytically in any case.

This hypotensive syndrome may result from the very high plasma concentrations of OP noted, which may reflect the relatively low lipid solubility of dimethoate (log K*ow* 0.76 (21)) (and therefore limited and slow tissue distribution) compared with other OP agents (5). However, as mentioned above, severe hypotension has been reported in some patients reporting ingestion of highly fat-soluble OPs such as fenthion (log K*ow* 4.30 (21)) and fenitrothion (log K*ow* 3.37 (21)). Our experience in Sri Lanka is that severe hypotension is rare in fenthion poisoning (5). If the syndrome is largely because of the persistent very high blood concentrations in dimethoate poisoning, we would predict it to also occur commonly with methamidophos and oxydemeton methyl, both OPs with expected limited tissue distribution due to low log K*ow* values less than 1.0 (21).

Alternatively, the toxicity may reflect co-formulants in the EC (22) preparation. These compounds include emulsifiers or surfactants, and organic solvents such as cyclohexanone and xylene. The cardiotoxic effect of the surfactants used in glyphosate herbicide is the best recognized example of co-formulant toxicity (23,24). However, the situation with organo-phosphorus EC insecticides is less clear with a sparse literature on their co-formulants. Furthermore, each company has its own formula for the EC preparation using different (often patented) solvents and emulsifiers.

The solvent is unlikely to be responsible for the dimethoate-specific syndrome as the majority of generic OP insecticide products available in Sri Lanka use 40% xylene as their solvent base (5). In particular, the solvent does not usually differ between chlorpyrifos and dimethoate despite their different syndromes. We have also found a very clear relationship between plasma dimethoate concentration and outcome in Sri Lankan patients (25). As the patients have ingested different brands of dimethoate, with different emulsifiers and other co-formulants, it seems unlikely that they can be primarily responsible for dimethoate EC40’s toxicity. Animal experiments to directly address the relative toxicity of dimethoate active ingredient and formulated dimethoate insecticide will likely be needed to answer the question and are currently underway (Eddleston & Clutton, unpublished).

Possible pathophysiological mechanisms include a primary cardiotoxic effect (26) or peripheral vasodilatation causing a distributive shock (14) or a mixed picture. We saw no evidence of direct cardiotoxicity – the cholinergic bradycardia was readily reversed with atropine, there was no rise in troponin T, and a Holter monitor in one patient showed normal sinus rhythm until just before death. ECGs taken from cases 1 and 2 showed no QT or QRS prolongation. Fluid loss did not play a major role – atropine rapidly reversed the muscarinic features of poisoning, controlling fluid loss. All patients continued to pass urine at good rates until death.

Animal studies have demonstrated multiple mechanisms of toxicity that may lead to hypotension in acute OP poisoning. OPs cause peripheral vasodilatation via the effect of acetylcholine on muscarinic receptors on vascular endothelium. They may also block nicotinic transmission at sympathetic and parasympathetic ganglia, leading to inhibition of the baroreceptor reflexes. Animal studies have also suggested that chronic OP exposure may cause oxidative damage of the vascular endothelium (27). One study has suggested that mast cell-mediated effects (anaphylaxis) may occur after OP poisoning (28), potentially leading to histamine-induced hypotension.

The patients presented here were treated in under-resourced hospitals similar to the hospitals that treat most OP cases globally. Resources for direct measurement of SVR and cardiac output were not available. However, clinical observation suggested that peripheral vasodilatation and distributive shock are likely to be the main mechanisms. The patients had marked peripheral vasodilatation on admission (Fig. 1) despite severe hypotension that was not apparently linked with right heart failure. We were struck by the relative brady-cardia that accompanied the marked hypotension, likely resulting from overwhelming cholinergic stimulation and therefore slowing of the heart in the context of reduced SVR. Treatment with atropine reversed the bradycardia (Fig. 2).

Buckley and colleagues (14) previously recommended giving very high doses of atropine, rather than inotropes, to treat OP-induced hypotension. However, case 3 (and many other cases in the cohort (5)) received over 200 mg of atropine soon after admission without effect on their BP. Unfortunately, norepinephrine was not available at this hospital, as studies of septic shock suggest that it might have been effective when the patients became refractory to dopamine (29). However, Asari and colleagues (16) found no effect of norepinephrine administration in four OP cases. Similarly, Kamijo and colleagues (15) found no benefit from a dopamine infusion in their patient and were forced to initiate extracorporeal life support. Case 3 in this series died despite large doses of epinephrine but he was so unwell on arrival to hospital that it was unlikely that he would have responded to any combination of inotropes.

Large case series with proven exposure to other OPs are required to determine whether the syndrome occurs with other pesticides and whether the extent of hypotension is inversely proportional to the lipid solubility. Future studies must include direct assessment of cardiac output and SVR in previously fit patients to define this syndrome more accurately and improve management. Further evidence is also required on the effectiveness of vasopressors and anticholinergic drugs.
